# Effects of Latent Solvent Content on Tuning the Nanofiltration Performance of Nanofibrous Composite Membranes

**DOI:** 10.3390/membranes15040118

**Published:** 2025-04-08

**Authors:** Xu-Dong Cao, Yu-Xuan Shao, Qian Wang, Tian-Dan Lu, Jing Zhong

**Affiliations:** 1 Jiangsu Key Laboratory of Advanced Catalytic Materials & Technology, School of Petrochemical Engineering, Changzhou University, Changzhou 213164, China; 2 National-Local Joint Engineering Research Center of Biomass Refining and High-Quality Utilization, Institute of Urban and Rural Mining, Changzhou Key Laboratory of Biomass Green, Safe & High Value Utilization, Changzhou University, Changzhou 213164, China

**Keywords:** membrane structure regulation, nanofiber, electrospinning, liquid separation

## Abstract

This study aims to optimize the application of electrospun nanofibrous substrates in thin-film composite (TFC) nanofiltration (NF) membranes for enhanced liquid separation efficiency by employing a method of effective welding between fibers using latent solvents. Polyacrylonitrile (PAN) nanofiber substrates were fabricated via electrospinning, and a dense polyamide selective layer was formed on their surface through interfacial polymerization (IP). The investigation focused on the effects of different solvent systems, particularly the role of dimethyl sulfoxide (DMSO) as a latent solvent, on the nanostructure and final membrane performance. The results indicate that increasing the DMSO content can enhance the greenness of the fabrication process, the substrate hydrophilicity, and the mechanical strength, while also influencing the thickness and morphology of the polyamide layer. At a DMSO rate of 30%, the composite membrane achieves optimal pure water permeability and high rejection rates; when the DMSO content exceeds 40%, structural inhomogeneity in the substrate membrane leads to an increase in defects, significantly deteriorating membrane performance. These findings provide theoretical insights and technical guidance for the application of electrospinning technology in designing efficient and stable NF membranes.

## 1. Introduction

Nanofiltration (NF) has emerged as a key technology within the field of pressure-driven membrane separation, commanding attention in a variety of industrial applications [1,2]. Functioning between ultrafiltration and reverse osmosis, NF has a distinctive screening mechanism, attributable to the charge conferred upon the selective layer by its constitutive materials and specific fabrication processes, which has proven essential in many applications, from complex pharmaceutical processes to comprehensive liquid treatment [3,4]. Commercial NF membranes predominantly feature a thin-film composite (TFC) structure, characterized by polyamide chemistry. These membranes are commonly constructed with a dense polyamide rejection layer atop a porous substrate [5]. Although the separation performance of TFC membranes is predominantly governed by the properties of their ultrathin polyamide layer, the porous sublayer significantly impacts the interfacial polymerization process and plays a crucial role in determining the overall performance characteristics of the membrane [6,7].

In the fabrication of porous sublayers, electrospinning emerges as a key technique. Electrospun nanofibrous membranes (ENMs) benefit from highly porous structures that enhance permeability and mechanical strength, thereby optimizing the membrane’s separation efficiency [8]. However, when electrospinning technology is applied to the manufacturing of separation membranes, it is crucial that the nanofibers exhibit good structural stability to withstand the shear forces and expansion issues encountered during exposure to fluids [9]. High-temperature treatment [10] and solvent vapor post treatment to enable fiber resorption are common methods. These methods require the precise control of the processing conditions to avoid adverse effects on the membrane structure. Another effective method to enhance the structural integrity and mechanical strength of polymer nanofiber membranes is to use non-invasive techniques to reinforce the connection points within the loosely structured nanofiber mat, allowing the nanofibers to fuse only at the junctions between fibers [11]. This process can lead to partial fusion between fibers, enhancing their connectivity and overall membrane strength. 

Unlike conventional phase inversion techniques, the pores in nanofiber membranes are created through fiber accumulation, with solidification taking place prior to deposition on the collector [12]. Consequently, the disintegration process of the electrospinning jet dictates the foundational dimensions of the resultant membrane structure. This highlights the critical role of jet disintegration dynamics in determining the structural characteristics of nanofiber-based composite membranes [13]. 

A multitude of factors during the electrospinning process determine the fiber structure, encompassing solution properties, environmental conditions, and process parameters. Notably, the solvent properties and polymer characteristics are particularly crucial in defining the electrospun fiber’s structure and its subsequent processability [14,15]. The phase separation rate, governed by solvent volatility and polymer–solvent interactions, determine spinnability. Excessively rapid phase separation can cause nozzle blockage, hindering continuous spinning, while overly slow phase separation may prevent the jet from overcoming the surface tension in order to split properly, or fail to solidify post deposition on the collector, resulting in dense membrane structures. In addition, optimizing and improving the greenness of solvents also plays an important role in the sustainable development of electrospinning processes.

Herein, we introduce a welding method that balances polymer–solvent interactions with solvent properties, employing latent solvents to enhance nanofiber performance. This approach enables the effective regulation of composite membrane characteristics, optimizing both structural integrity and functional properties, while also offering essential insights into the impact of nanofiber support properties on NF performance and presenting practical approaches for constructing high-performance TFC NF membranes.

## 2. Materials and Methods

### 2.1. Materials 

Polyacrylonitrile (PAN, MW: 80,000 Da) was used as the raw material to prepare nanofibrous substrates. N,N-dimethylformamide (DMF, ≥99.5%, Lingfeng Chemicals, Shanghai, China), N,N-dimethylacetamide (DMAc, ≥99.5%, Lingfeng Chemicals, Shanghai, China), dimethyl sulfoxide (DMSO, ≥99.5%, Lingfeng Chemicals, Shanghai, China), and N-methyl-2-pyrrolidone (NMP, ≥99.5%, Lingfeng Chemicals, Shanghai, China) were used as solvents for the membrane preparation. Piperazine (PIP, AR, Aladdin, Hangzhou, China) was used as the amine monomer in the aqueous phase, and trimesoyl chloride (TMC, ≥99%, Aladdin, Hangzhou, China) was used as the acyl chloride monomer for interfacial polymerization (IP). N-hexane (≥97%, AR, Aladdin, Hangzhou, China) was used as a solvent. Na_2_SO_4_, NaCl, MgCl_2_, and MgSO_4_ were purchased from Sinopharm Chemical Reagent Co., Ltd, and used as a testing target solute. Polyethylene glycol (PEG) of different molecular weights (PEG 200, PEG 400, PEG 800, PEG 1000, PEG 2000) was purchased for membrane pore size distribution characterization. The deionized (DI) water was produced using a homemade water purifier.

### 2.2. Preparation of Support Layers

The preparation of PAN nanofiber substrates: The PAN nanofiber mats were fabricated via the electrospinning process under an optimized electrical field. A 10 wt% solution of PAN was prepared in either a single solvent or a mixture of solvents. This solution underwent electrospinning through a needle with a diameter of 0.6 mm. The polymer solution was delivered at a steady flow rate of 15 μL/min. A high-voltage power supply established a potential difference of 25 kV between the needle tip and a cylindrical collector, positioned 12 cm apart. The resulting nanofiber mat was collected using a roller receiver equipped with a grounded metal roller shaft, operating at a speed of 100 rpm. After collection, the as-spun nanofiber mats were carefully detached from the aluminum foil and subsequently hot pressed at 80 °C under a pressure of 0.025 MPa for a specified duration to achieve a smooth surface suitable for the deposition of upper layers. Following this, the fibrous membrane was placed in a convection drying oven at 80 °C for 12 h to remove any residual solvent, resulting in a membrane thickness of 130−170 μm.

### 2.3. Preparation of TFC Membranes

A thin selective layer was prepared on the surface of the nanofiber substrates via interfacial polymerization (IP) between PIP and TMC solutions. Prior to the reaction, the PI substrates obtained in the first step were washed with deionized water three times to eliminate the influence of residue solvent inside nanofibers and stored in deionized water. The substrate was then fixed on an interface polymerization frame, and a certain amount of 2.0 wt% PIP aqueous solution was gently poured onto the surface of the substrate. After standing for 2 min, the aqueous phase solution was poured out, and the residual aqueous phase solution on the surface was wiped off with a wiping paper and a rubber roller. Then, a certain amount of 0.1 wt% TMC/n-hexane solution was poured onto the membrane surface and reacted for 1 min before pouring out the excess organic phase solution. Finally, the prepared thin composite membrane was washed three times with n-hexane and stored in deionized water for later use.

### 2.4. Characterization

A rotary viscometer (NDJ-8S, Shanghai Fangrui Co., Ltd., Shanghai, China) was used to measure the viscosity of spinning solution samples. The morphological structures of membranes were examined using a scanning electron microscope (SEM, Hitachi Regulus 8100, Suzhou, Japan). The membranes were first freeze-dried before being sputter-coated with gold. For observation of the cross-sections, the membrane was first immersed in anhydrous ethanol before being fractured in liquid nitrogen. The hydrophilicity of the membrane surface was characterized through static contact angle measurements, employing the sessile drop mode. A 2 μL droplet of deionized water was dropped onto the sample surface. Once the droplet structure stabilized, the contact angle at the liquid–solid interface was measured using a contact angle analyzer (OSA 100, Ningbo NB Scientific Instrument Co., Ltd., Ningbo, China). The results were averaged from three values achieved at different locations of one membrane sample. A tensile testing machine (WDT Series, Shenzhen KQI Test Equipment Co., Ltd., Shenzhen, China) was used to measure the mechanical properties of PAN ENMs. The dried samples were customized into rectangular shapes with dimensions of 5 mm × 10 cm, and then clamped vertically between the two sample racks of the testing machine. The thickness of the sample was measured using an electronic micrometer with an accuracy of ±0.1 μm. The stretching speed was set at 5 mm/min. The membrane samples were prepared as 1.0 × 1.0 cm, washed repeatedly with pure water, and freeze-dried.

### 2.5. Evaluation of Membrane Performance

Typically, the membrane separation performance includes pure water permeability (PWP) and rejection rate. A homemade cross-flow filtration device was used to measure the separation performance. Experiments for testing the support layer permeances were conducted at 1 bar and 4 bar for TFC membranes, both under room temperature. Before each test, each membrane was pre-treated with deionized water for at least 30 min, to ensure that the performance reached a stable state. Then, 0.1 g/L dye (Congo red, MW = 696.68 Da) or 1 g/L salt solution was used as a feeding solution. The permeability can be calculated using Equation (1):

(1) $$P=\frac{V}{A\Delta t\Delta p}$$

where P is the permeance (L m^−2^h^−1^bar^−1^) and V is the volume of the permeate (L). A is the filtration area (m^2^), Δt is the filtration time (h), and Δp is the pressure (bar). 

The rejection (R) of solutes was calculated using the following Equation (2):

(2) $$R=\left(1-\frac{c_{p}}{c_{f}}\right)\times 100\%$$

where *c*_p_ and *c*_f_ are the salt concentrations of permeate and feed solutions, respectively. The concentrations of salt and dye were measured using a conductivity meter (Thunder Magnetic DDS-307, LABO-HUB, Shanghai, China) and an UV-Vis spectrophotometer (UV-1900, himadzu Europa GmbH, Duisburg, Germany), respectively. 

To test the pore size distribution of TFC membranes, PEG solutions with a concentration of 0.2 g/L were utilized as feed solutions at a transmembrane pressure of 6 bar, respectively. The effective solutes rejection R (%) was calculated using Equation (3). The concentrations of PEG were tested using a total organic carbon analyzer (Vario TOC, Elementar, Elementar Analysensysteme GmbH, Langenselbold, Germany). The relationship between solute rejection (RT) and diameter (u) can be described using a log–normal probability function:

(3) $$R_{T}=\mathrm{erf}\left(y\right)=\frac{1}{\sqrt{2\pi }}\int_{-\infty }^{y}e^{-u^{2}/2}du$$

where

(4) $$y=\frac{\ln r_{s}-\ln\mu_{s}}{\ln\theta }$$

and *r*_s_ is the solute radius, *μ*_s_ represents the mean solute radius at *R_T_* = 50%, and *θ*_p_ is the standard deviation of *μ*_s_. In the log–normal probability graph, Equations (3) and (4) can be described in a linear formation.

(5) $$F\left(R_{T}\right)=A+B\left(lnr_{s}\right)$$

Finally, the pore size distribution can be calculated using Equation (6):

(6) $$\frac{dR_{T}\left(r_{p}\right)}{dr_{p}}=\frac{1}{r_{p}\ln\theta_{p}\sqrt{2}}exp\left[-\frac{{\left(lnr_{p}-ln\mu_{p}\right)}^{2}}{2{\left(\ln\theta_{p}\right)}^{2}}\right]$$

## 3. Results

During the high-pressure solution electrospinning process, the solution forms a highly stretched and viscoelastic ultrafine jet under the action of the electrostatic field, which solidifies either during the jetting process or on the collector, resulting in reoriented and aligned polymer molecular chains. Generally, the properties of the solution, including solubility and rheological and electrical properties, can significantly affect the morphology and separation performance of the nanofibrous membrane. Meanwhile, the greenness of the spinning solution is of vital importance for the electrospinning process. In this work, we compared common spinning solvent systems, aiming to enhance the greenness of the system while retaining a stable spinning process. Additionally, we sought to effectively regulate the content of latent solvents in the solidified nanofibers to achieve control over the membrane morphology during post-treatment processes. Among the most commonly used solvents for dissolving PAN, DMF, DMSO, DMAc, and NMP were selected for investigation.

### 3.1. Selection of Electrospinning Solvents

The solvent plays a role in dispersing the polymer molecular chains in the spinning solution. To design and determine the optimal electrospinning solvent system, it is necessary to conclude the affinity between the polymer and the selected solvent. This determines the electrical and rheological characteristics of the spinning solution, which in turn influence the structural and morphological features of the formed nanofibers, ultimately affecting the filtration performance of the membranes. The interaction distance (Ra) can reflect the polymer–solvent affinity; a smaller Ra value displays a good polymer–solvent compatibility and also represents a slower phase transition rate. Ra can be calculated using the following equation, where the subscript s refers to the solvent and subscript p refers to the polymer, *D* stands for disperse force, *P* stands for polar force, *H* stands for hydrogen bonding force (the corresponding parameters is provided in Table 1):

(7) $$Ra=\sqrt{{4\left(\delta_{D,p}-\delta_{D,s}\right)}^{2}+{\left(\delta_{P,p}-\delta_{P,s}\right)}^{2}+{\left(\delta_{H,p}-\delta_{H,s}\right)}^{2}}$$

From the Table 1, it can be seen that the order of Ra is as follows: DMAc > DMF > NMP > DMSO, indicating that DMSO obtains the best affinity with PAN. In the electrospinning process, fiber splitting and solidification determine the fiber morphology, so the fundamental properties of the solvent, such as the dielectric constant, volatility, and solubility of the polymer in the solvent, all have an impact on the structure and morphology of the electrospun fibers. The solvent’s dielectric constant determines its ability to carry charge in an electrostatic field and affects the jet splitting stability, whilst also impacting the fiber uniformity. The saturation vapor pressure of the solvent and the solvent–polymer affinity jointly determine the volatility of the solvent and the stage of complete fiber solidification. Table S1 presents the fundamental solvent properties, revealing that DMF exhibits the higher saturation vapor pressure, indicating strong volatility. DMSO is noted for possessing a relatively higher dielectric constant [17].

### 3.2. Green Degree Analysis

Due to the critical importance of solvent utilization in the electrospinning process, it is important to explore relatively environmentally friendly or green solvents from the perspective of environmental protection and green production. The GlaxoSmithKline (GSK) solvent sustainability guide is designed to offer a comprehensive comparison of individual sustainability criteria, incorporating a composite score for ranking purposes by integrating multiple facets of sustainability. It consists of five categories: waste assessment score, environmental impact assessment score, health assessment score, safety assessment score, and life cycle assessment score. For each assessment category, an overall summary score is defined as the geometric mean of each of the relevant category scores [18]:

(8) $$Waste=\sqrt[{4}]{I\times R\times BT\times VOC}$$

(9) $$Enviroment=\sqrt{Air\times Aqueous}$$

(10) $$Health=\sqrt{Health\;hazard\times Exposure\;potential}$$

(11) $$Safety=\sqrt{F\&E\times R\&S}$$

where *I*, *R*, *BT*, *VOC*, *F&E*, and *R&S* are the incineration score, recycle score, biotreatment score, VOC emissions score, flammability and explosion potential score, and reactivity and stability score, respectively. A higher score represents a higher greenness level. Additionally, a composite score is defined as the geometric mean of the waste, environmental impact, health, and safety scores, according to the following equation:

(12) $$Composite=\sqrt[{4}]{Waste\times Eniromental\times health\times safety}$$

The detailed calculations for each category and composite score are shown in Table 2. It can be concluded from the table that the DMSO obtains the highest greenness score. In order to achieve the effective regulation of the fiber-phase transition rate, DMF with high evaporability and DMSO with lower Ra are suitable as a solvent in electrospinning solution systems, as they differ significantly in terms of polymer solubility, dielectric constant, and saturation vapor pressure. 

### 3.3. Mixed Solvent Regulation of Basic Properties of Fiber Membrane

For the DMSO/DMF mixed solvents, each category score is calculated based on a weight algorithm using the following equation:

(13) $${Score}_{mix}=x_{1}\times {Score}_{1}+y_{2}\times {Score}_{2}$$

where x_1_ and y_2_ are the volume fractions of DMSO and DMF in mixture solvents, respectively, and Score1 and Score2 are each category score of DMSO and DMF, respectively.

**Figure 1:**
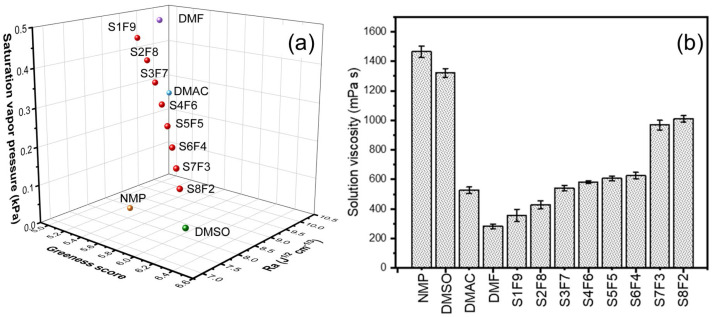
(**a**) The saturated vapor pressure, Ra, and composite GSK greenness score of different solvents and DMSO/DMF systems in a three-dimensional box. (**b**) Effect of solvent system on the viscosity of 10 wt% PAN spinning dope. The used DMSO/DMF mixture solvents with different volume ratios (VDMSO: VDMF = x/y) were defined as SxFy, respectively.

The saturated vapor pressure, Ra, and the greenness score of the mixed solvent are detailed in Table S2 and Figure 1a, where it can be seen that with the increase in the DMSO fraction, the Ra and saturated vapor pressure decrease synchronously, which stands for a lower phase transition rate during the fiber stretch. In addition, with the enhancement of the volume ratio of DMSO/DMF, the corresponding GSK composite score increases and surpasses other solvents when the DMSO rate is higher than 20 vol.%. This reveals that DMSO/DMF systems are greener alternatives for regulating the electrospinning process due to the high waste and environment and safety values of DMSO (Table S3). As shown in Figure 1b, the solid content is fixed in 10 wt% for the study of the solution’s properties, especially for the effect of the DMSO solvent ratio on the viscosity of the spinning solution. With the increase in the DMSO content, the viscosity of the spinning solution continuously increases, making it more difficult and slower to extend the jet [19]. It is difficult for spinning to proceed under high solution viscosity surpassing 800 mPa s.

**Figure 2:**
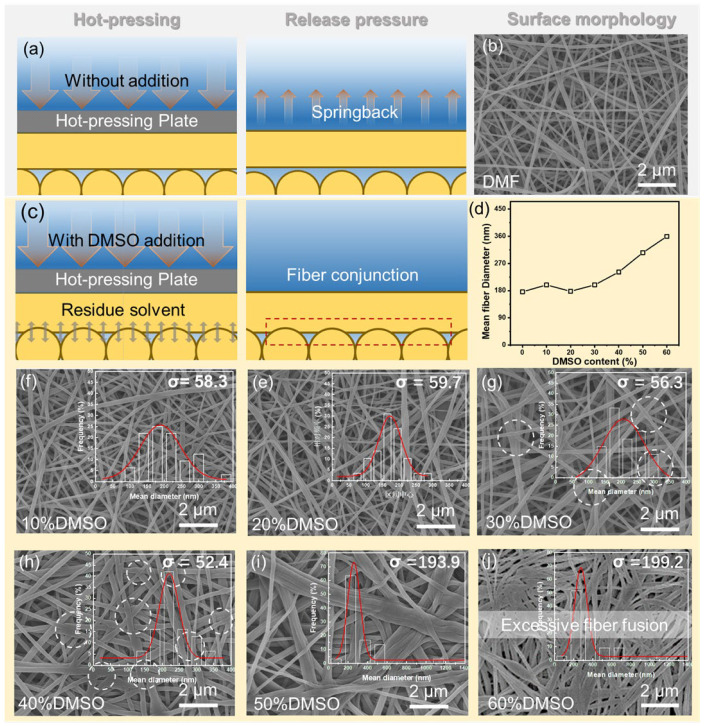
Schematic illustration of the electrospun nanofibrous substrate fabrication process using (**a**,**b**) a single DMF solvent system and (**c**) a mixed solvent system. (**d**) Influence of solvent composition on the average fiber diameter; (**e**−**j**) SEM images of the surface morphology of the electrospun nanofibrous substrates from blended solvents and fiber diameter distribution.

Based on the above results, further investigations involved the SEM characterization of the surface morphology of nanofiber membranes after hot-press treatment, aiming to elucidate the effect of solvents on the morphology of the fiber membranes, and the results are shown in Figure 2. Observations revealed that at DMSO contents below 20 vol.%, the surface of the fiber membrane exhibited a relatively loose felt-like structure with no apparent bonding points between fibers. As the DMSO content increased, the surface of the fiber membrane gradually became smoother, and effective welding points among fibers started to appear. The number of these welding points increased with the rise in the DMSO content. This phenomenon was attributed to the latent solvent in the polymer network. During the subsequent hot-press treatment, the elevated temperature further promoted the increase in the saturated vapor pressure, which facilitated the re-dissolution process from inside the fibers to the outside. This mechanism strengthened the connections between nanofibers (Figure 2c).

The fiber diameter distribution chart shows that with the increase in the DMSO ratio, the diameter distribution of nanofibers (represented by the standard deviation σ) initially becomes more uniform. When the mass fraction of DMSO exceeds 40 vol.%, the range of the fiber diameter distribution gradually widens, indicating excessive welding and fusion among fibers, reducing structural uniformity. According to Table S2, as the proportion of DMSO increases, the dielectric constant of the mixed solvent also continuously increases, which leads to a decrease in the polarization effect of the electrostatic field on the polymer solution, thereby increasing the average size of polyacrylonitrile nanofibers (Figure 2d) [20]. 

**Figure 3:**
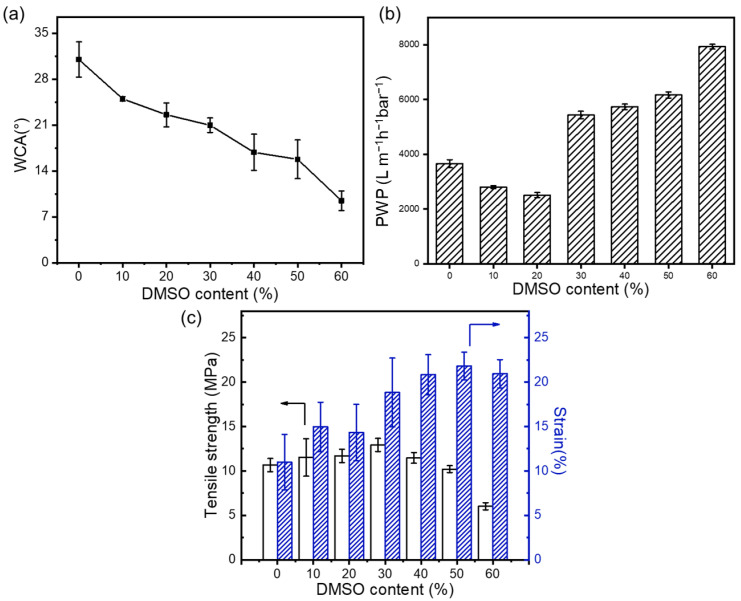
Effect of mixed solvent ratio on (**a**) the hydrophilic properties of the support layer, (**b**) the pure water permeance, and (**c**) on the mechanical properties of the nanofibrous support layers.

Figure 3a illustrates that with the increase in the DMSO content, the hydrophilicity of the membrane significantly enhances due to the microstructural changes in the nanofibers caused by the introduction of DMSO, which can amplify the wetting performance of the membrane surfaces. As can be concluded from Figure 3b, when the DMSO content is below 20%, the pure water flux (PWP) of the membrane is primarily governed by variations in the membrane pore structure. As the DMSO content increases, the high hydrophilicity of the membrane begins to dominate the substrate permeance change, thereby enhancing the membrane flux. This indicates that at lower DMSO concentrations, adjustments in the pore structure play a crucial role in permeability. However, with increasing DMSO content, the enhanced hydrophilicity significantly contributes to improved water affinity and permeation through the membrane, leading to an increase in flux. This highlights the importance of solvents in optimizing the performance of nanofibrous membranes for advanced filtration performance. The welding among fibers also impacts the mechanical strength of the substrate. The influence of the DMSO content on the mechanical strength of nanofibrous membranes is shown in Figure 3c. The elongation at the break of nanofiber-based membranes steadily increases, while the maximum tensile stress slightly increases with the addition of DMSO. This is mainly attributed to the increased welding points among fibers, which reduce the interlayer slippage and thereby enhance mechanical stability. However, further increasing the DMSO content beyond 30 vol.% leads to a decrease in the maximum tensile stress, due to uneven fiber size distribution causing stress defects. This trend indicates that controlling the DMSO content in spinning solutions can effectively adjust the mechanical properties of nanofibrous membranes, highlighting the importance of precise formulations in achieving the desired material characteristics.

### 3.4. The Influence of Substrate Structure on the Performance of TFC Membranes

**Figure 4:**
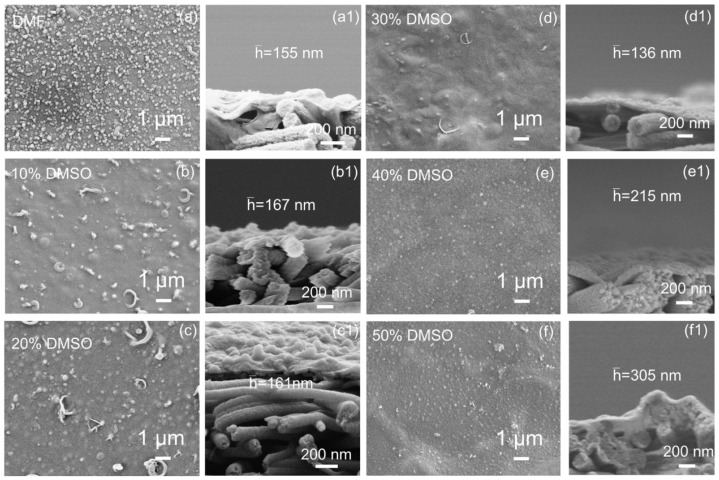
SEM morphology of (**a**−**f**) surface and (**a1**−**f1**) cross-section of polyamide TFC membranes based on nanofibrous substrates.

To explore the impact of nanofibrous substrate membrane structures on interfacial polymerization, thin-film composite membranes were fabricated. The substrate’s surface pore size significantly affects this process and the polyamide (PA) layer’s morphology. As shown in Figure 4, using DMF as the sole solvent for electrospinning results in nanosphere nodules spread across the membrane, typical of a classical PA layer. Elevating latent solvent content leads to thinner separation layers, resulting in larger vesicle sizes and fewer vesicle numbers. This occurs due to the restricted membrane pore sizes and reduced monomer diffusion across the membrane [21]. Polymer fibers’ effective “welding” and smaller pores limit the selective layer’s infiltration. Notably, when DMSO exceeds 30 vol.%, the separation layer thickens again due to a more hydrophilic membrane surface, enhancing water phase interaction and PA layer formation. These observations underscore the importance of substrate properties in optimizing the structure and performance of PA selective layers in composite membranes.

**Figure 5:**
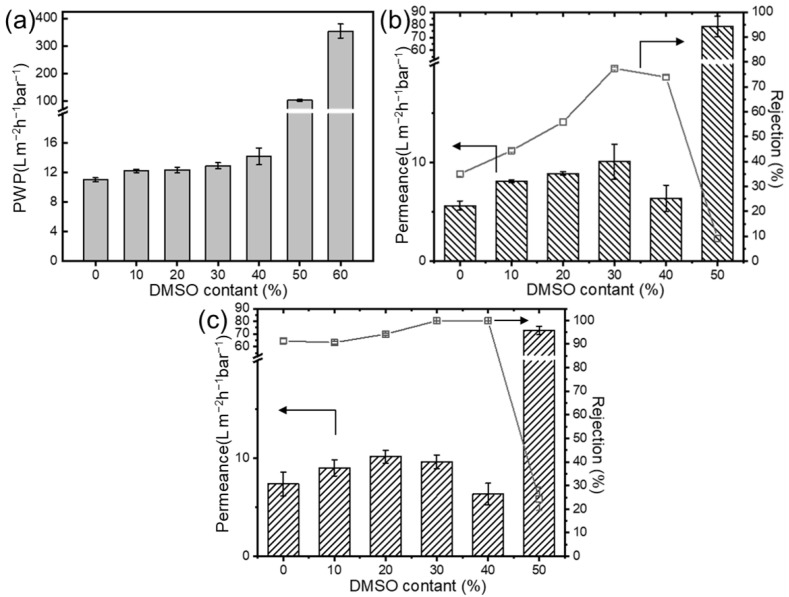
Effect of mixed solvent ratio on (**a**) the pure water permeance; rejection performance (**b**) to salt, and (**c**) dye solution of TFC membrane (Axis breaks are used here to avoid unnecessary blank spaces in the diagram).

The performance changes in the composite membranes were evaluated using salt and dye solutions. Figure 5 illustrates the influence of nanofiber substrate membrane structure on the performance of composite membranes. The pure water permeability (PWP) significantly increased with the higher DMSO content, which is due to the smaller substrate pore size reducing the selective layer penetration in the substrate. The solution flux initially rises and then declines, which is due to the more severe membrane fouling resulting from looser selective layer pore sizes. Conversely, the rejection rate remained at a relatively constant high, then drastically decreased at higher DMSO concentrations. When the DMSO content exceeds 40 vol.%, the structural inhomogeneity of the substrate membrane leads to an increase in defects, significantly deteriorating the separation layer’s integrity and causing a notable decline in the performance and deactivation of the composite membranes. These results indicate an optimal DMSO concentration of approximately 30%, which achieves high permeability and acceptable rejection rates (99.9% to Congo red).

**Figure 6:**
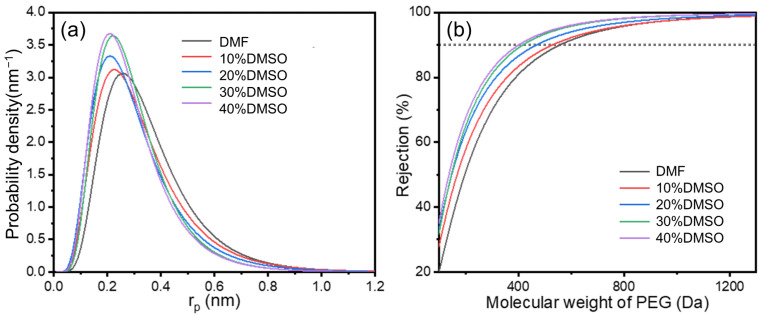
(**a**) The pore size distribution and (**b**) MWCO curves of TFC membranes.

The pore size results of optimized composite membranes are shown in Figure 6 and Table 3, calculated based on the rejection results of neutral solutes, and the pore size distribution was obtained using the log−normal distribution model between the solute rejection rate and the solute Stokes radius. These results further demonstrate the structural changes in the selective layer. With an enhancement of the DMSO ratio, the MWCO of the PA separation layer decreases. Table 3 illustrates the influence of as-prepared TFC membranes with varying DMSO ratios on the mean effective pore radius and standard deviation. The mean effective pore radius of TFC membranes generally decreases. The pore size distribution corresponds to the trend observed in the nanofiber size distribution, highlighting the significant role of the substrate structure in controlling the selective layer.

## 4. Conclusions

This study comprehensively compared the solubility parameters and solvent properties of commonly used strong polar aprotic solvents with PAN, among which the DMF/DMSO system is more suitable for achieving a greener process as well as the effective regulation of fiber structures. The dissolution characteristics of the polymer in mixed solvents, along with changes in the dielectric constants and saturated vapor pressure, exert staged impacts on the nanofiber membrane structure. By adjusting the solvent ratio, the effective welding degree between fibers increased with the rising latent solvent content in the solidified fibers, enhancing the mechanical strength of the substrate membrane and reducing the WCA. The resultant TFC membranes were prepared, and their performance was compared based on different mixed solvent systems. The results indicate a high correlation between fiber structure and final TFC membrane performance; increasing the latent solvent ratio improves the composite membrane separation performance. However, excessive welding leads to a decrease in the substrate membrane pore uniformity, causing defects in the selective layer and reducing the rejection performance.

## Figures and Tables

**Table 1:** Solubility parameters and interaction distances of PAN polymer and solvents [16].

Materials	*δ*_D_ (J^1/2^ cm^−1/3^)	*δ*_P_ (J^1/2^ cm^−1/3^)	*δ*_H_ (J^1/2^ cm^−1/3^)	Ra (J^1/2^ cm^−1/3^)
PAN	21.7	14.1	9.1	-
DMSO	18.4	16.4	10.2	7.08
NMP	18	12.3	7.2	7.85
DMF	17.4	13.7	11.3	8.89
DMAc	16.8	11.5	10.2	10.20

**Table 2:** Scores for solvents based on GSK solvent sustainability guide [19].

Solvent	Waste				Environment		Human Health		Safety		Life Cycle Analysis	Composite Score
	Incineration	Recycling	Biotreatment	VOC Emissions	Aqueous Impact	Air Impact	Health Hazard	Exposure Potential	Flammability and Explosion	Reactivity and Stability		
DMSO	3	4	4	9	8	6	7	9	9	5	6	6.40
NMP	3	4	3	10	10	6	1	9	9	9	4	5.49
DMAC	3	6	3	9	10	6	1	7	9	9	2	5.42
DMF	3	6	3	8	10	4	1	6	9	9	7	5.37

**Table 3:** Mean effective pore radius and standard deviation of TFC membranes.

DMSO Ratio (vol.%)	r_p_ (nm)	Standard Deviation
0	0.32	1.58
10	0.29	1.65
20	0.27	1.66
30	0.27	1.56
40	0.26	1.56

## Data Availability

The raw data supporting the conclusions of this article will be made available by the authors on request.

## Supplementary Materials

The following supporting information can be downloaded at: https://www.mdpi.com/article/10.3390/membranes15040118/s1. Table S1: The properties of selected solvents and viscosity of 10 wt% PAN solution; Table S2: The properties of DMSO/DMF mixture solvents system; Table S3: Category scores and composite scores for DMSO/DMF mixture solvents based on GSK solvent sustainability guide.
