# Supplementary material for: Effects of Latent Solvent Content on Tuning the Nanofiltration Performance of Nanofibrous Composite Membranes
Source: Membranes (Basel). 2025 Apr 8;15(4):118. doi: 10.3390/membranes15040118 (PMC12029555; doi:10.3390/membranes15040118)
Supplement: Supplementary file 1 [file membranes-15-00118-s001.zip › membranes-3546925-supplementary.pdf]

Supporting Information

Effects of Latent Solvent Content on Tuning the Nanofiltration Performance of Nanofibrous-Based Composite Membranes

Xu-dong Cao[a], Yu-xuan Shao[a], Tian-dan Lu[a]*, Jing Zhong[a]*

a. Jiangsu Key Laboratory of Advanced Catalytic Materials & Technology, School of Petrochemical Engineering, Changzhou University, Changzhou, 213164, PR China

Table.S1 The properties of selected solvents and viscosity of 10wt% PAN solution

Solvent	Relative dielectric constant (25 °C)	Boiling point (°C)	Saturated vapor pressure(kPa,25 °C)
**NMP**	33.7	202	0.04
**DMAc**	37.8	166	0.26
**DMF**	36.7	153	0.50
**DMSO**	46.7	189	0.09

Source: ASPEN PLUS Property Database.

Table.S2 The properties of DMSO/DMF mixture solvents system

Solvent	δ_D	δ_P	δ_H	R_a	Relative dielectric constant (25 °C)	Saturated vapor pressure (kPa,25 °C)
**S1F9**	17.5	16.67	11.19	9.03	37.7	0.453
**S2F8**	17.6	16.64	11.08	8.81	38.7	0.408
**S3F7**	17.7	16.61	10.97	8.59	39.7	0.363
**S4F6**	17.8	16.58	10.86	8.37	40.7	0.321
**S5F5**	17.9	16.55	10.75	8.15	41.7	0.279
**S6F4**	18	16.52	10.64	7.94	42.7	0.239
**S7F3**	18.1	16.49	10.53	7.72	43.7	0.200
**S8F2**	18.2	16.46	10.42	7.50	44.7	0.162

Table.S3 Category scores and composite scores for DMSO/DMF mixture solvents based on GSK solvent sustainability guide

Solvent	Waste	Environment	Health	Safety	Composite score
**S1F9**	4.56	6.38	3.00	8.77	5.26
**S2F8**	4.56	6.45	3.55	8.54	5.46
**S3F7**	4.56	6.51	4.10	8.31	5.64
**S4F6**	4.56	6.57	4.64	8.08	5.79
**S5F5**	4.56	6.63	5.19	7.85	5.92
**S6F4**	4.56	6.69	5.74	7.62	6.04
**S7F3**	4.56	6.75	6.29	7.40	6.15
**S8F2**	4.56	6.81	6.84	7.17	6.25
